# DeerLab: a comprehensive software package for analyzing dipolar electron paramagnetic resonance spectroscopy data

**DOI:** 10.5194/mr-1-209-2020

**Published:** 2020-10-01

**Authors:** Luis Fábregas Ibáñez, Gunnar Jeschke, Stefan Stoll

**Affiliations:** 1 Laboratory of Physical Chemistry, ETH Zurich, Vladimir-Prelog-Weg 2, 8093 Zurich, Switzerland; 2 Department of Chemistry, University of Washington, Seattle, WA 98195, USA

## Abstract

Dipolar electron paramagnetic resonance (EPR) spectroscopy (DEER and other techniques) enables the structural characterization of macromolecular and biological systems by measurement of distance distributions between unpaired electrons on a nanometer scale. The inference of these distributions from the measured signals is challenging due to the ill-posed nature of the inverse problem. Existing analysis tools are scattered over several applications with specialized graphical user interfaces. This renders comparison, reproducibility, and method development difficult. To remedy this situation, we present DeerLab, an open-source software package for analyzing dipolar EPR data that is modular and implements a wide range of methods. We show that DeerLab can perform one-step analysis based on separable non-linear least squares, fit dipolar multi-pathway models to multi-pulse DEER data, run global analysis with non-parametric distributions, and use a bootstrapping approach to fully quantify the uncertainty in the analysis.

## 1 Introduction

Dipolar electron paramagnetic resonance (EPR) spectroscopy encompasses a growing family of techniques for determining distributions of nanometer-scale distances between unpaired electrons. These distance distributions provide valuable information for the structural characterization of macromolecular or biological systems that is complementary to information obtained by other techniques. For structurally disordered or highly complex systems, where established techniques may fail, such distance distributions provide unique information. The family of dipolar EPR spectroscopy techniques includes double electron–electron resonance (DEER) [Bibr bib1.bibx60], double quantum coherence (DQC) [Bibr bib1.bibx70], relaxation-induced dipolar modulation enhancement (RIDME) [Bibr bib1.bibx52], single-frequency technique for refocusing (SIFTER) [Bibr bib1.bibx44], and several other related techniques [Bibr bib1.bibx7]. All of them provide a time-domain signal that depends on the dipolar interaction between pairs of electrons. From this time-domain signal, the distance distribution is inferred.

Due to its mathematical nature, the robust inference of distance distributions from noisy dipolar EPR spectroscopy data is not straightforward. Many approaches have been proposed to tackle this problem [Bibr bib1.bibx64], each with its pros and cons. Some of these methods have found widespread use, via software packages such as DeerAnalysis [Bibr bib1.bibx47], GLADD/DD [Bibr bib1.bibx9], and LongDistances [Bibr bib1.bibx2].

However, there are several major challenges with the current situation. (i) A comparative assessment of the relative merits of various methods is missing. (ii) Many methods have been argued based on anecdotal evidence from small datasets, and their performance has not been assessed comprehensively. (iii) Reproducibility of analysis results is very limited due to the lack of common platforms for data sharing and data analysis.

To remedy this situation, we introduce DeerLab, an open-source software for data analysis in dipolar EPR. It is based on the Python programming language and consists of a collection of modular functions, analogous to EasySpin [Bibr bib1.bibx78] and Spinach [Bibr bib1.bibx36]. This has several distinct advantages over a graphical user interface (GUI). (i) It allows for very flexible workflow designs, easily adapting to different experimental situations. (ii) All existing methods can be directly compared on a single platform. (iii) Automation and processing of large datasets become straightforward. (iv) Scripted data analysis improves reproducibility and collaboration. (v) It provides a foundation for implementing new methodologies. (vi) It can be embedded into other software, such as tools for protein structure modeling based on distance distributions [Bibr bib1.bibx43]. The disadvantage is an accessibility barrier for potential users without programming skills. This disadvantage can be remedied by building a dedicated GUI for standard workflows as an additional software layer.

This paper is structured as follows. We start by summarizing the theoretical basics of dipolar EPR spectroscopy. Then, we illustrate the functionality of DeerLab through a series of examples. First, we show how to reproduce well-established workflows such as Tikhonov regularization and multi-Gauss fits. We then demonstrate how DeerLab can perform one-step analysis based on separable non-linear least-squares optimization, fit dipolar multi-pathway models to multi-pulse DEER data, and run global analysis with non-parametric distributions. Finally, several sections are dedicated to important aspects of uncertainty analysis, including the use of bootstrapping. The DeerLab scripts for generating all figures, as well as the corresponding distance distributions and dipolar signals, are available in the supporting information.

## 2 Theoretical basics

This section summarizes the central theoretical concepts of dipolar EPR spectroscopy that DeerLab is based on. For more details, see [Bibr bib1.bibx41]. The theory is limited to S=1/2 spins with isotropic g-values, without any orientation selection, at most two spins per protein, no exchange coupling, weak dipolar coupling, and no conformer-dependent relaxation rates.

Dipolar EPR spectroscopy techniques measure the magnetic dipole–dipole couplings between spins via the modulation of the amplitude V(t) of a spin echo as a function of the position t of one or more pump pulses. The echo amplitude is modeled as [Bibr bib1.bibx60]

1 $$V(t)=V_{0}\cdot V_{\mathrm{intra}}(t)\cdot V_{\mathrm{inter}}(t),$$

where $V_{0}$ is the echo amplitude in the absence of any pump pulses, $V_{\mathrm{intra}}$ describes the pump-pulse-induced modulation due intra-molecular dipolar couplings, and $V_{\mathrm{inter}}$ describes the modulation due to intermolecular couplings. $V_{0}$ is a constant prefactor that we set to one from now on in order not to complicate the notation unnecessarily. DeerLab takes $V_{0}$ into account as a fitting parameter.

The product of intra- and inter-molecular dipolar modulations can be written in a general form as

2 $$V(t)=\int_{0}^{\infty }K(t,r)P(r)\;dr.$$

P(r) is the distribution of intra-molecular spin–spin distances r on the protein or other complex, normalized such that $\int_{0}^{\infty }P(r)dr=1$.

K(t,r) is the kernel that captures how the complete dipolar modulation is determined by the distance distribution. It includes the inter-molecular modulation [Bibr bib1.bibx27]. For standard four-pulse DEER it is

3 $$K(t,r)=\left[\left(1-\lambda )+\lambda K_{0}(t,r\right)\right]V_{\mathrm{inter}}(t,\lambda ).$$

Here, λ is the modulation depth. $K_{0}$ is the elementary kernel

4 $$K_{0}(t,r)=\int_{0}^{1}\mathrm{cos}\left[(1-3{\mathrm{cos}}^{2}\theta )Dr^{-3}t\right]d\mathrm{cos}\theta ,$$

with the dipolar coupling constant

5 $$D=\frac{\mu_{0}}{4\pi }\frac{g_{e}^{2}\mu_{B}^{2}}{\hbar },$$

where $g_{e}$ is the g-value of the free electron, $\mu_{B}$ the Bohr magneton, $\mu_{0}$ the magnetic constant, and ℏ the reduced Planck constant. $K_{0}$ assumes full orientation averaging and unlimited excitation bandwidth. The subscript 0 distinguishes this elementary kernel from more general kernels such as Eq. ([Disp-formula Ch1.E3]).

$V_{\mathrm{inter}}(t,\lambda )$ represents the inter-molecular modulation and is commonly called the background. It can be modeled as a stretched-exponential function

6 $$V_{\mathrm{inter}}(t,\lambda )=\mathrm{exp}\left(-\kappa_{d}\lambda |t{|}^{d/3}\right),$$

where $\kappa_{d}$ is a decay rate constant and d is the dimensionality [Bibr bib1.bibx53]. Other background models are possible [Bibr bib1.bibx50].

Experimentally, the echo amplitude is measured only for a discrete set of usually equally spaced time points $t_{i}$, yielding a dipolar signal vector V with n elements $V_{i}=V(t_{i})$. For numerical analysis, P(r) is represented as a discrete distance distribution vector P with m elements $P_{j}=P(r_{j})$ at equally spaced $r_{j}$. With this, Eq. ([Disp-formula Ch1.E2]) reads

7 V=KP,

where K is the n×m kernel matrix with elements $(K{)}_{ij}=K(t_{i},r_{j})\Delta r$, and Δr is the increment in the distance domain.

Experimental data $V_{\exp}$ deviate from in Eq. ([Disp-formula Ch1.E1]) due to presence of noise. From experiments, it was found that the noise distribution in DEER signals is well approximated by an uncorrelated Gaussian distribution with zero mean and constant variance [Bibr bib1.bibx23]:

8 $$V_{\exp}=V+N\left(0,\sigma^{2}I\right).$$

Inferring the distance distribution from the dipolar signal formally requires inversion of the kernel matrix

9 $$P=K^{-1}V_{\exp}.$$

However, K is generally badly ill-conditioned (it has an extremely large condition number). This renders the inverse problem ill-posed, and the results obtained by Eq. ([Disp-formula Ch1.E9]) are highly unstable, erratic, and unreliable, especially in the presence of noise. Because of the ill-posedness, inferring distance distributions from the dipolar signals poses a major challenge in dipolar EPR spectroscopy data analysis.

## 3 Current approaches

Currently, two families of methods are commonly used in dipolar EPR spectroscopy data analysis. They differ in whether the distance distribution is represented as a parametric model or as a non-parametric model. Both methods stabilize the solution and are widely used since they are simple and often effective.

In the following, we discuss these two families of methods from the perspective of DeerLab.

### 3.1 Non-parametric distributions

If P is represented as a non-parametric vector, regularization methods are used to determine the solution. If background and modulation depth are known and fixed, the associated regularized optimization problem has the form

10 $$P_{\mathrm{fit}}=\underset{P\geq 0}{\mathrm{argmin}}\left({∥V_{\exp}-KP∥}^{2}+\alpha^{2}R(LP)\right).$$

The first term represents the sum of squared residuals, i.e., $\chi^{2}$ without normalization by the noise variance, which we assume constant across the signal (see Eq. [Disp-formula Ch1.E8]). It quantifies the quality of the fit of the model to the data. The second term is an additional penalty term that, together with the non-negativity constraint P≥0, stabilizes the solution. The regularization matrix L is a numerical approximation of a differential operator to impose smoothness, and α is the regularization parameter, which controls the balance between data agreement and regularization.

Regularization methods differ in the choice of the penalty norm R. Tikhonov regularization [Bibr bib1.bibx82] was the first regularization approach introduced for dipolar data analysis [Bibr bib1.bibx8]. Other approaches such as total variation (TV), Huber regularization, and Osher's Bregman-iterated regularization are beneficial in certain cases [Bibr bib1.bibx26].

In the absence of the non-negativity constraint, the problem in Eq. ([Disp-formula Ch1.E10]) could be solved directly by $P_{\mathrm{fit}}=\bar{K}V_{\exp}$, with the α-dependent regularized pseudoinverse of the kernel matrix

11 $$\bar{K}={\left(K^{T}K+\alpha^{2}L^{T}L\right)}^{-1}K^{T}$$

in the case of Tikhonov regularization. However, due to the non-negativity constraint, this is not possible, and Eq. ([Disp-formula Ch1.E10]) is solved using non-negative least-squares optimization algorithms [Bibr bib1.bibx54]. $\bar{K}$ will be useful for uncertainty analysis (see Sect. [Sec Ch1.S9]).

The selection of α has been optimized for a large test set in previous work [Bibr bib1.bibx24] and replicated [Bibr bib1.bibx26], revealing that selection methods such as the Akaike information criterion (AIC) [Bibr bib1.bibx1] or general cross-validation (GCV) [Bibr bib1.bibx32] can be superior to L-curve criteria [Bibr bib1.bibx33] if Gaussian white noise is the only source of error.

DeerLab provides a flexible regularization framework including all the aforementioned α-selection, non-iterated and iterated regularization methods, as well as a selection of solvers. In particular, implementation of Huber and TV regularization is much improved compared to our original study [Bibr bib1.bibx26], such that these now perform similarly to Tikhonov regularization, invalidating the conclusions on the under-performance of Huber and TV regularization from that initial work.

Figure [Fig Ch1.F1] shows how regularization approaches can be compared using DeerLab. Figure [Fig Ch1.F1]a presents an example of a low-noise dipolar signal processed via Tikhonov, TV and Osher's Bregman (Tikhonov) iterated regularization. All methods using the AIC for α-selection yield similar results. Figure [Fig Ch1.F1]b illustrates how regularization methods perform in the presence of strong noise. In such cases more differences arise between the different methods.

**Figure 1 Ch1.F1:**
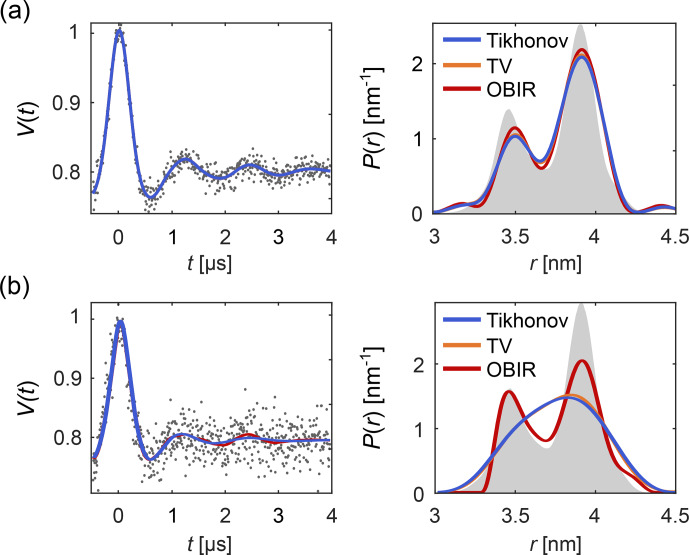
Analysis of background-free dipolar signals with regularization. A distance distribution is fit to **(a)** a low-noise signal and **(b)** a high-noise signal using Tikhonov regularization (blue), TV regularization (orange), and Osher's Bregman-iterated (OBIR) regularization (red). In all cases, the AIC was used for the selection of the regularization parameter (see Fig. S1). The input data (simulated) are shown as grey dots and the ground truth distance distribution is shown as grey shaded areas.

The outcomes of regularization analysis depend strongly on the choice of penalty norm, regularization operator, and α. For the remainder of this work, if not specified otherwise, we will use the Tikhonov penalty equipped with the second-order difference operator $L_{2}$ and the AIC for α-selection.

### 3.2 Parametric models

In an alternative representation, P is described as a parametric model P[θ] with $\left(P[\theta ]{)}_{i}=P(t_{i},\theta \right)$, where θ is a vector of a small number of parameters. This is fit to the data using

12 $$\theta_{\mathrm{fit}}=\underset{\theta }{\mathrm{argmin}}{∥V_{\exp}-KP[\theta ]∥}^{2}.$$

Since solving the inverse problem by fitting a non-linear parametric model with only a few parameters is a well-conditioned problem, it can be solved without the need for regularization.

While bimodal Gaussian distributions were the first parametric models [Bibr bib1.bibx64], the idea was generalized to a linear combination of N Gaussian distributions [Bibr bib1.bibx73] (which we will refer to as a multi-Gauss model)

13 $$P[\theta ]=\sum_{i=1}^{N}a_{i}p_{i}[{\bar{r}}_{i},\sigma_{i}],$$

where $a_{i}$ are the amplitudes and $p_{i}$ are the normalized Gaussian basis functions parameterized by their center distances ${\bar{r}}_{i}$ and widths $\sigma_{i}$. Other parameterizations of the amplitudes can be used [Bibr bib1.bibx9].

To determine the optimal number N of Gaussians in the multi-Gauss model, [Bibr bib1.bibx73] proposed a statistical F-test, while [Bibr bib1.bibx77] and [Bibr bib1.bibx38] introduced the corrected Akaike information criterion (AICc) [Bibr bib1.bibx79] and the Bayesian information criterion (BIC) [Bibr bib1.bibx72]. Two more recent approaches utilize Monte Carlo simulations to determine the optimal multi-Gauss model [Bibr bib1.bibx22]. Parametric models are not limited to Gaussian basis functions. Many other basis function types (or mixtures thereof) can be employed, e.g., 3D Rice distributions [Bibr bib1.bibx21], spherical distributions [Bibr bib1.bibx39], random-coil models [Bibr bib1.bibx29], or worm-like chain models [Bibr bib1.bibx86].

In a milestone for parametric modeling, Brandon et al. expanded the use of parametric models to include the modulation depth and a stretched-exponential background in the analysis of the signal in their software GLADD/DD [Bibr bib1.bibx9]. This results in a time-domain parametric model

14 $$V[\theta ]=V[\lambda ,\theta_{P},\theta_{B}]=K[\lambda ,\theta_{B}]P[\theta_{P}].$$

In this, the parameter vector θ includes not only the distance distribution parameters $\theta_{P}$, but also the modulation depth λ and the background parameters $\theta_{B}$. In general, a parametric time-domain model can be fit to the experimental data by solving

15 $$\theta_{\mathrm{fit}}=\underset{\theta }{\mathrm{argmin}}{∥V_{\exp}-V[\theta ]∥}^{2}.$$

DeerLab expands upon this, allowing the design and fitting of any kind of time-domain or distance distribution parametric model. Automated multi-Gauss fitting and model selection using AIC, BIC, and other metrics are provided as well. In Fig. [Fig Ch1.F2] we provide such an example of multi-Gauss fitting with DeerLab. The signal is fit using the time-domain model Eq. ([Disp-formula Ch1.E14]) and a varying number of Gaussians as the distance distribution model. Model selection based on the AIC determines the most parsimonious number of Gaussians, with a decent fit of the distance distribution.

**Figure 2 Ch1.F2:**
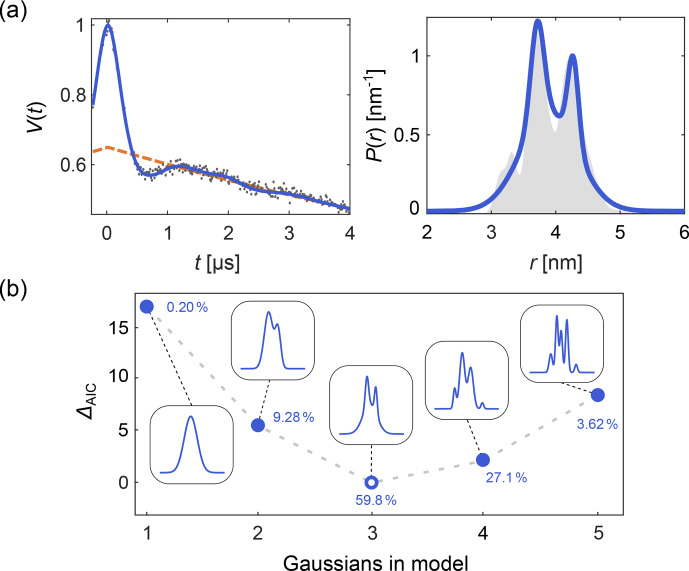
Time-domain multi-Gauss fitting of a simulated four-pulse DEER signal. **(a)** The data are given as grey dots, the ground truth distance distribution as a shaded area, and the corresponding fits of a 3-Gauss model as blue lines. The background fit is given as dashed orange line. **(b)** The difference in AIC values as a function of the number of Gaussians is shown in blue, and the corresponding fits are given in the insets. The differences $\Delta_{\mathrm{AIC}}$ are relative to the model with the lowest AIC value. The corresponding Akaike weights (see Sect. [Sec Ch1.S10]) are given next to each model. The model with the lowest AIC value (i.e., largest Akaike weight) is selected as the optimal model.

It is, however, crucially important to keep in mind that approaches based on parametric models may suffer from selection bias, i.e., the bias introduced by limiting the analysis to a specific family of models and by using a particular criterion for model selection within that family [Bibr bib1.bibx30]. Parametric model fits may also be affected by confirmation bias, i.e., the tendency to process data in a way that matches one's preconceptions and avoids contradiction of prior belief [Bibr bib1.bibx63].

## 4 One-step analysis

Neither parametric model fitting nor regularization is ideal. Parametric models can be fit directly to the full time-domain signal but are strongly limited by how well they can approximate the ground truth distribution. On the other hand, regularization yields non-parametric distance distributions that accommodate a much larger range of ground truths, but regularization cannot directly include other parameters such as the background and the modulation depth.

With regularization methods, it is therefore common practice to use a two-step approach: (1) fit a parametric background model to the time-domain signal and correct the signal by the fitted background (either by division or subtraction) and (2) apply regularization to the background-corrected signal. Software based on this approach includes DeerAnalysis [Bibr bib1.bibx47] and LongDistances [Bibr bib1.bibx2]. Including the fitted background in the kernel for step (2), as in Eq. ([Disp-formula Ch1.E3]), also does not eliminate the need for the two-step approach [Bibr bib1.bibx27].

The two-step analysis as done in DeerAnalysis is sub-optimal, because the background fit in step (1) relies on the assumption that the oscillation periods in the time-domain signal are much shorter than the overall signal length. Many experimentally observed signals do not satisfy this assumption. Therefore, the two-step analysis cannot robustly process these types of signals.

The most desirable approach is to simultaneously fit both the time-domain parameters θ and a non-parametric distance distribution P to the time-domain signal $V_{\exp}$ in one step, i.e.,

16 $$\left(\theta_{\mathrm{fit}},P_{\mathrm{fit}}\right)=\underset{\theta ,P\geq 0}{\mathrm{argmin}}F(\theta ,P),$$

with the regularized objective function

17 $$F(\theta ,P)={∥V_{\exp}-K[\theta ]P∥}^{2}+\alpha^{2}R(LP).$$

Equation ([Disp-formula Ch1.E16]) can be solved using a variety of constrainable non-linear optimization algorithms by combining θ and P into a single parameter vector and applying all the constraints for θ and P [Bibr bib1.bibx2]. However, this does not take full advantage of the special structure of the problem, i.e., that it is a penalized least-squares problem with a model for V that is linear in P and non-linear in θ.

To take advantage of this structure, DeerLab implements a nested subspace optimization algorithm based on separable non-linear least-squares [Bibr bib1.bibx13]. It separates the θ and P spaces and uses a non-linear least-squares algorithm to solve

18 $$\theta_{\mathrm{fit}}=\underset{\theta }{\mathrm{argmin}}F\left(\theta ,P[\theta ]\right),$$

where P[θ] is the optimal non-parametric distance distribution for a given θ, determined via regularization

19 $$P[\theta ]=\underset{P^{\prime }\geq 0}{\mathrm{argmin}}F\left(\theta ,P^{\prime }\right)$$

as in Eq. ([Disp-formula Ch1.E10]), using a dedicated non-negative linear least-squares algorithm. (Note that P is now a parametric model since it depends on θ, although this includes only modulation depth and background parameters.) Once $\theta_{\mathrm{fit}}$ is obtained, $P_{\mathrm{fit}}$ is calculated as $P[\theta_{\mathrm{fit}}]$. The algorithm is iterative and is illustrated in Fig. [Fig Ch1.F3]. It starts with an initial guess for θ and determines the associated P from Eq. ([Disp-formula Ch1.E19]). This P is then used by the algorithm of Eq. ([Disp-formula Ch1.E18]) to determine the next θ, which is then used again by the algorithm in Eq. ([Disp-formula Ch1.E19]) to get the next P[θ], and so on until convergence is reached. The optimization in Eq. ([Disp-formula Ch1.E19]) can utilize any form of regularization, and it can be run with a fixed regularization parameter α or optimize it each time.

**Figure 3 Ch1.F3:**
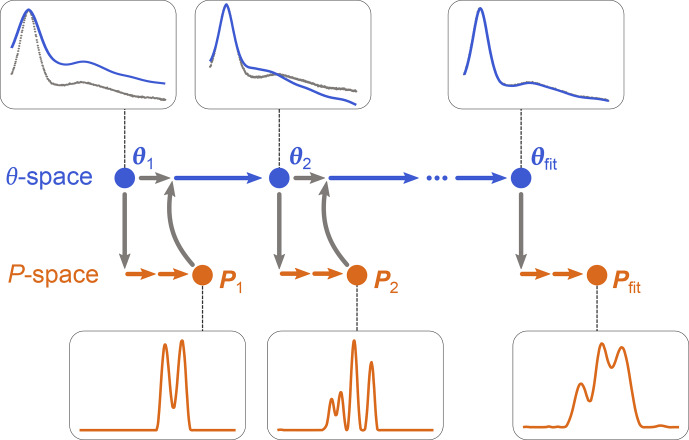
One-step analysis of dipolar signals using separable non-linear least-squares optimization. A set of parameters $\theta_{n}$ (blue) is computed by optimization of Eq. ([Disp-formula Ch1.E18]) for some given experimental signal $V_{\exp}$ (grey dots). For each $\theta_{n}$, a corresponding distance distribution $P_{n}$ (orange) is computed by optimization of Eq. ([Disp-formula Ch1.E19]). This procedure is repeated until a minimum of the objective function is found for an optimal parameter set $\theta_{\mathrm{fit}}$, leading to the corresponding optimal distribution $P_{\mathrm{fit}}$.

**Figure 4 Ch1.F4:**
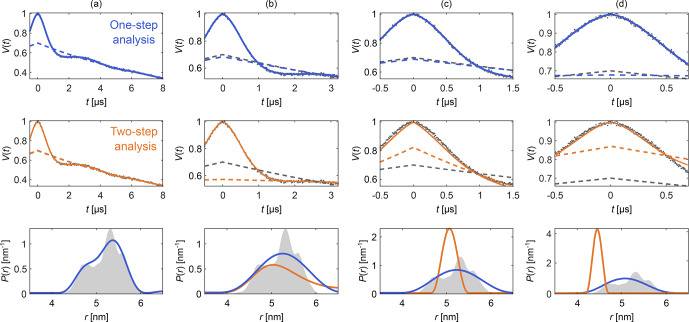
Comparison of one-step and two-step analysis. A stretched-exponential background and a non-parametric distance distribution were fit to a simulated four-pulse DEER signal. The analysis was done by either fitting both simultaneously (blue) or by fitting the background followed by the distribution (two-step analysis, orange). The analysis was repeated on the same signal with increasing truncation **(a–d)**. The two-step analysis fails in case **(d)**. The data are given as grey dots, and the fitted signal and background are given as solid and dashed colored lines, respectively. The true background is given as a grey dashed line for reference. The non-parametric distance distributions obtained via Tikhonov regularization are given as, respectively, colored lines and the ground truth as a shaded area.

Figure [Fig Ch1.F4] shows an example that compares this one-step approach to the traditional two-step analysis, using progressively more truncated dipolar signals. Analogous comparisons between two-step analysis and fully parametric models have been previously reported [Bibr bib1.bibx9]. For a sufficiently long signal (Fig. [Fig Ch1.F4]a) both approaches yield similar results, as the signal is long enough for the oscillations to decay, facilitating the separate fit of the background in the two-step analysis. If the signal is truncated as in Fig. [Fig Ch1.F4]b–d, the two-step analysis cannot properly fit the background anymore. In contrast, one-step analysis correctly identifies the background in all cases and recovers the underlying non-parametric distance distribution with reasonable fidelity. It is important to keep in mind that highly truncated signals such as the ones shown in Fig. [Fig Ch1.F4]d can fail to provide correct results if the measurement noise is too large (see Fig. S2 in the Supplement).

This shows that DeerLab's ability to simultaneously fit the background and a non-parametric distribution opens up the possibility of fitting non-parametric distance distributions to signals which might have been deemed unanalyzable in the past.

## 5 Multi-pathway models

Dipolar EPR spectroscopy found widespread use with four-pulse DEER. Since then, experimental dipolar EPR spectroscopy has developed into a set of diverse techniques, with signals exhibiting a variety of features. However, the data processing has not evolved much from its four-pulse DEER origins.

The key operator in the analysis is the dipolar kernel K(t,r) (see Eq. [Disp-formula Ch1.E2]). It represents the experiment; i.e., it describes how the time-domain signal is obtained from a given distance distribution. If the kernel cannot account for a feature in the signal, it is because the kernel model is incomplete. In these cases, it is preferable to improve the kernel rather than to tweak the signal into an artificial four-pulse DEER signal.

One example of this is RIDME, where it is known that the measured signals in S>1/2 systems contain overtones not present in four-pulse DEER [Bibr bib1.bibx67]. If disregarded, these overtones cause distortions in the distance distribution if the four-pulse DEER kernel is used to analyze the data. This can be avoided by including the overtones in the model [Bibr bib1.bibx51]. In DeerLab, one can include a set of overtones with a background in the kernel model, which can be used to directly fit primary RIDME data via, e.g., regularization.

Other examples are multi-pulse DEER sequences, which generally are not fully modeled. All multi-pulse DEER experiments feature modulations in addition to the basic modulation of Eq. ([Disp-formula Ch1.E3]). Despite their clear dipolar origin, these are regarded as undesirable “artifacts”: the “2+1 artifact” in four-pulse DEER [Bibr bib1.bibx41] and “artifacts” or “residues” in five-pulse and seven-pulse DEER [Bibr bib1.bibx7]. Several experimental and processing approaches have been published that aim to remove these contributions from the total signal to recover the idealized dipolar evolution function [Bibr bib1.bibx7]. These approaches introduce further experimental or theoretical complexity.

However, these additional contributions are actual dipolar signals. They may even provide strong oscillations at times when the oscillations from the main signal have decayed, thus increasing the signal-to-noise ratio of the experiment. Instead of removing these contributions due to the lack of a proper model, it is advantageous to extend the model to explicitly include these contributions.

DeerLab includes such an extended model for multi-pulse DEER, derivable from spin density matrix dynamics. In this model, which we call the dipolar multi-pathway model, the overall signal is a combination of several dipolar signals arising from dipolar pathways of varying amplitudes and refocusing times [Bibr bib1.bibx69]. The total signal is given by Eq. ([Disp-formula Ch1.E2]) with the general kernel

20 $$\begin{array}{rl} K(t,r) & =\left[\Lambda_{0}+\sum_{p=1}^{N}\lambda_{p}K_{0}(n_{p}(t-T_{p}),r)\right]\\ & \cdot \prod_{p=1}^{N}V_{\mathrm{inter}}\left(n_{p}\left(t-T_{p}\right),\lambda_{p}\right), \end{array}$$

where $\Lambda_{0}$ is the total contribution of the unmodulated dipolar pathways, the index p runs over all N modulated dipolar pathways, $T_{p}$ are the refocusing times of the individual modulated dipolar pathways, $\lambda_{p}$ are the amplitudes of the modulated dipolar pathways, $n_{p}$ are the harmonics of the individual modulated pathways, and the background function is as in Eq. ([Disp-formula Ch1.E6]). A schematic representation is shown in Fig. [Fig Ch1.F5]. The kernel for standard four-pulse DEER from Eq. ([Disp-formula Ch1.E3]) is a special case of Eq. ([Disp-formula Ch1.E20]), with N=1, $T_{1}=0$, $n_{1}=1$ $\Lambda_{0}=1-\lambda$, and $\lambda_{1}=\lambda$.

**Figure 5 Ch1.F5:**
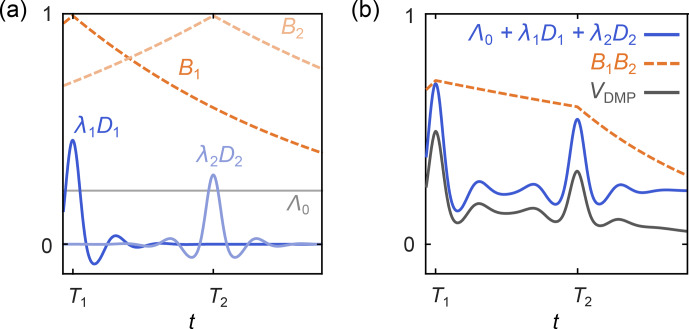
Schematic representation of a dipolar multi-pathway DEER signal. **(a)** Background decays (orange dashed lines) and dipolar evolution functions (blue solid lines) are shown for two different dipolar pathways. The unmodulated component $\Lambda_{0}$ is given as a solid grey line. **(b)** The overall signal (black) is given by the sum over all pathway dipolar evolution functions (blue) times the product over all pathway background decays (orange).

Discretization of the kernel in Eq. ([Disp-formula Ch1.E20]) gives the same expression as in Eq. ([Disp-formula Ch1.E7]),

21 V=K[θ]P,

with the parameter set θ comprising $\Lambda_{0}$, all $\lambda_{p}$, $n_{p}$, $T_{p}$, as well as $\kappa_{d}$ and d.

Figure [Fig Ch1.F6] shows examples of how DeerLab can analyze multi-pulse DEER data in terms of a multi-pathway model, thus alleviating the need for experimental correction protocols or signal pre-processing (beyond phase correction). Note that experimental schemes which generate multiple datasets with some pathways shifted with respect to each other [Bibr bib1.bibx10] can profit from global analysis (vide infra) to stabilize the accurate estimation of pathway parameters.

**Figure 6 Ch1.F6:**
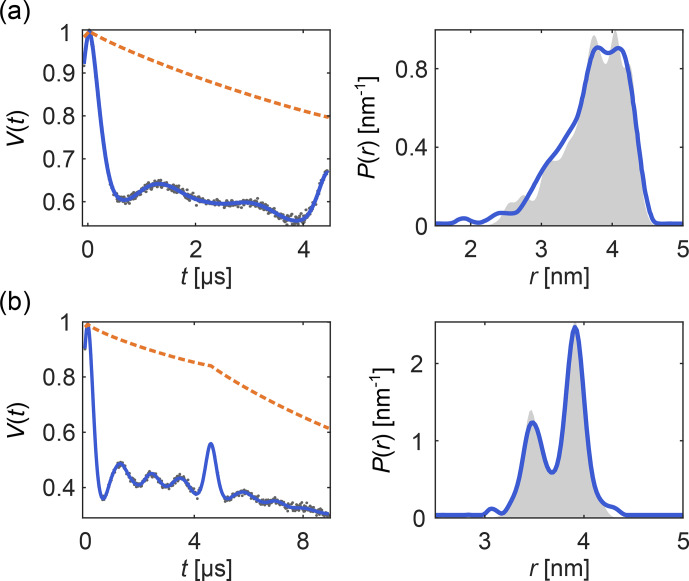
One-step analysis of multi-pulse DEER signals with the dipolar multi-pathway model for **(a)** four-pulse DEER with the “2+1 artifact” and **(b)** five-pulse DEER with its four-pulse “artifact”. All data were fit with two modulated dipolar pathways. The data are given as grey dots, the signal and distribution fits are given as solid blue lines, the background fit is given as an orange dashed line, and the ground truth is given as a shaded area.

This analysis of multi-pulse DEER signals shows the benefits of using full models for dipolar signals instead of removing or avoiding “artifacts” to try to match partial models. DeerLab provides a compact framework for testing, developing, and applying more complete models.

## 6 Global analysis

Global analysis denotes the situation where a single model is fit simultaneously to multiple datasets. In dipolar EPR, this was first used for fitting a multi-Gauss distribution model to several DEER datasets [Bibr bib1.bibx9]. Later, Tikhonov regularization was employed to fit short and long DEER traces simultaneously [Bibr bib1.bibx68].

Global analysis is implemented in DeerLab for both parametric and non-parametric distance distribution models and for an arbitrary number of dipolar signals. The global optimization problem of a set of M dipolar signals $V_{\exp,i}$ using a model depending on parameters θ and on N non-parametric distance distributions $P_{j}$ is

22 $$\left(\theta_{\mathrm{fit}},\{P_{\mathrm{fit}}\}\right)=\underset{\theta ,\{P\}\geq 0}{\mathrm{argmin}}\left[F(\theta ,\{P\})+G(\{P\})\right],$$

with $\left\{P\}=\{P_{1},\ldots ,P_{N}\right\}$ and

23F(θ,{P})=∑i=1Mwi‖Vexp,i-Viθ,{P}‖2σi2,24G({P})=α2∑j=1NR(LPj),

with a similar expression without {P} and G({P}) for a fully parametric model. $\sigma_{i}$ are the noise levels of the individual signals. The parameter vector θ includes the parameters needed to generate all signals $V_{i}$, where each $V_{i}$ typically depends only on a subset of the parameters.

The quantities $w_{i}$ in Eq. ([Disp-formula Ch1.E23]) are the weights that determine the contribution of each signal to the objective function. The default is $w_{i}=1$, meaning that each data point from each signal contributes equally, given its noise level, to the objective function. Different values of $w_{i}$ can be used to indicate preferential weighing.

If all signals $V_{i}$ derive from a single distance distribution, then we can use Eq. ([Disp-formula Ch1.E22]) together with

$$V_{i}[\theta ,P]=K_{i}[\theta ]P$$

or

25 $$V_{i}[\theta ]=K_{i}[\theta ]P[\theta ].$$

Here, each $K_{i}$ describes a different experiment (different pulse sequence, different trace length, etc.) and depends on a subset of the parameters in θ. The distance distribution P can be either parametric (in which case θ includes the distribution parameters) or it can be non-parametric. In the latter case, Eq. ([Disp-formula Ch1.E22]) is solved using separable non-linear least squares. As an example, in Fig. [Fig Ch1.F7] we simultaneously fit a four-pulse DEER signal and a five-pulse DEER signal with its secondary four-pulse pathway contribution, using a model with a single non-parametric distribution but separate backgrounds and modulation depths for the two signals. The distance distribution underlying both signals is nicely recovered.

**Figure 7 Ch1.F7:**
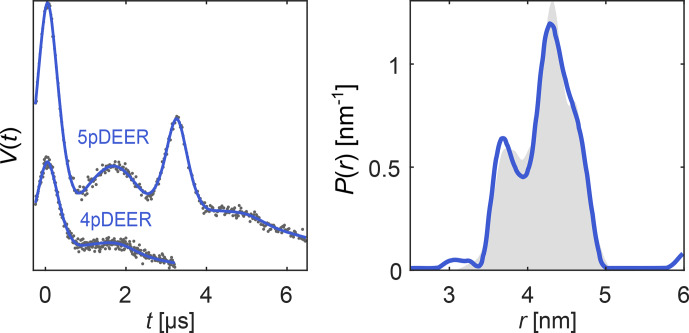
Global analysis of a four-pulse DEER signal and a five-pulse DEER signal with its secondary four-pulse pathway contribution, both derived from the same distance distribution. The simulated data are given as grey dots, the ground truth distribution is given as a shaded area, and the signal and distribution fits are given as solid blue lines.

Note, however, that the analysis of dipolar signals of different length or obtained under different dynamical decoupling conditions may be inconsistent if different conformers have different dephasing rates [Bibr bib1.bibx3].

Another common global analysis situation is when the measured signals stem from several samples containing a mixture of chemical or structural components, each with its own distinct distribution, and related to each other via additional conditions such as a chemical equilibrium. The simplest case is a system equilibrated between two forms A and B (A⇌B), e.g., a protein–ligand binding equilibrium or a monomer–dimer equilibrium. In this case, the dipolar signals are described as

26 $$\begin{array}{rl} V_{i}[\theta ,\{P\}] & =V_{i}[\{\theta_{K},x_{A}\},\{P_{A},P_{B}\}]\\ & =K_{i}[\theta_{K}]\left[x_{A,i}P_{A}+\left(1-x_{A,i}\right)P_{B}\right], \end{array}$$

with both component distributions $P_{A}$ and $P_{B}$ being fitted along the parameter set $\theta =\{\theta_{K},\{x_{A,i}\}\}$ via Eq. ([Disp-formula Ch1.E22]). The mole fractions $x_{A,i}$ depend on the location of the equilibrium, which might vary among the samples via ligand concentration, matrix composition, and other factors. Either the mole fractions or the underlying equilibrium constant can be included among the fitting parameters.

Such titration or dose–response datasets have been analyzed using multi-Gaussian distribution models for the component distributions [Bibr bib1.bibx77]. As discussed above, however, non-parametric distributions may often be a preferable choice. DeerLab enables global fitting of an arbitrary number of datasets with regularization approaches and, thus, analysis of titration datasets in terms of non-parametric distributions. As an example, Fig. [Fig Ch1.F8] shows such an analysis of a protein–ligand binding assay, using signals with different noise levels, trace lengths, backgrounds, and modulation depths (Fig. [Fig Ch1.F8]a) with a model that includes the bound-protein mole fractions among the parameters. The global analysis gives good fits to the time-domain data and results in two non-parametric distributions that capture the underlying ground truth well (Fig. [Fig Ch1.F8]b). The extracted mole fractions are consistent with the underlying binding equilibrium (Fig. [Fig Ch1.F8]c). Alternatively, one can skip the separate determination of the mole fractions and include the dissociation constant directly as a parameter in the global analysis of the dipolar data. Even in cases where one or both extremes of the binding curve are experimentally unavailable, such an analysis is still feasible albeit at the cost of larger uncertainty in the fitted molar fraction or dissociation constants. In summary, this example shows that DeerLab allows global analysis of titration datasets using non-parametric distributions.

**Figure 8 Ch1.F8:**
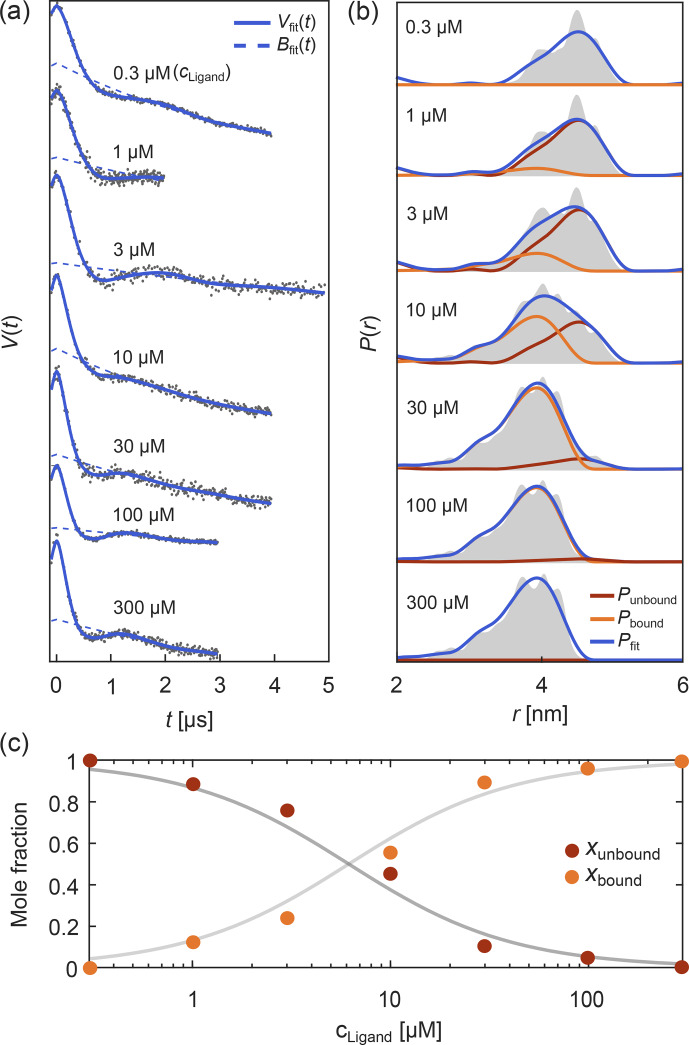
Global fitting of titration data of a protein–ligand binding equilibrium with non-parametric distributions. In panel **(a)** four-pulse DEER traces with different trace lengths, modulation depths, backgrounds, and noise levels were simulated for different ligand concentrations added to a protein concentration of 1 µM. These parameters as well as the mole fractions of bound/unbound protein for each trace and two non-parametric distance distributions for the bound and unbound states (via Tikhonov regularization) were fit simultaneously. The simulated data are given as grey dots and the fitted signals and backgrounds are given as solid and dashed blue lines, respectively. In panel **(b)** the distance distribution fits for the unbound (red) and bound (orange) states are given as well as the combined fitted distribution (blue) for the different ligand concentrations. The ground truth sum distributions are given as grey shaded areas. In panel **(c)** the fitted mole fractions of the unbound (red) and bound (orange) states are given as colored dots. The solid grey lines represent the ground truth for a dissociation constant of $K_{d}=5.65$ µM.

## 7 Global and local minima

Even when a fitted model agrees with the experimental data and a minimum of the objective function has been located, it does not mean that the only or the best fit has been found. The objective function can have multiple minima, meaning that the located minimum is not necessarily the global minimum (see Fig. [Fig Ch1.F9]). Which minimum is found depends mainly on where the search is started. The boundaries set on the parameter space can also influence the outcome if a minimum is located outside these boundaries. Other factors, e.g., the numerical algorithms and convergence parameters used for the optimization, can affect this as well.

**Figure 9 Ch1.F9:**
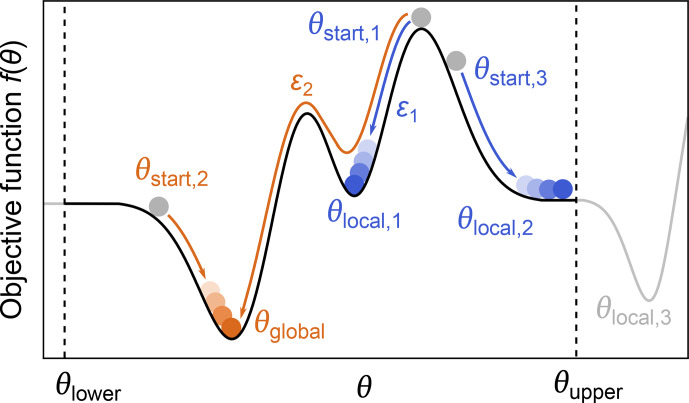
Global vs. local minima. During optimization of a parameter θ, by minimization of an objective function f(θ) (black line), several local minima (blue) might be found instead of the global minimum (orange). The global minimum can be found by varying the starting point, the lower/upper boundaries $\theta_{\mathrm{upper}}$ and $\theta_{\mathrm{lower}}$ (to find minima which might be outside the bounds, e.g., $\theta_{\mathrm{local},3}$), or the convergence parameters ε of the numerical solver such that some local minima might be ignored.

While there are dedicated global optimization algorithms, the simplest approach to find a global minimum is to repeat the optimization process with different starting values in order to explore the parameter space more fully [Bibr bib1.bibx73]. After sampling enough starting points, a set of minima is found and the one with the lowest objective function value is taken as the global minimum (see Fig. [Fig Ch1.F9]).

While these procedures can be costly, it is recommended to routinely check that variation of algorithmic parameters does not yield a lower minimum.

## 8 Goodness of fit

After obtaining a fitted model, an important step is to assess the goodness of the fit. If the fit is not good, either the optimization failed to locate an appropriate minimum (see previous section) or the model is inapplicable (e.g., oversimplified) for the given experimental data, and a better model needs to be used.

**Figure 10 Ch1.F10:**
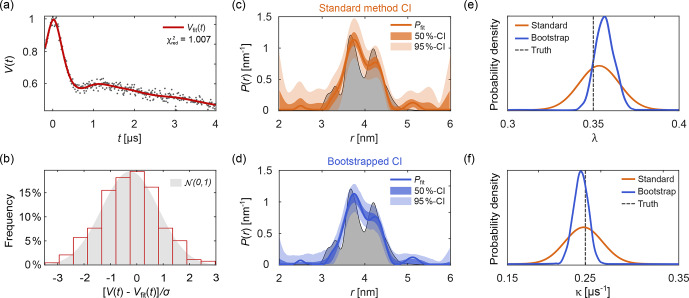
Uncertainty analysis. In panel **(a)** a simulated noisy four-pulse DEER signal with exponential background (grey dots) is fitted with a non-parametric distance distribution (red line). In panel **(b)**, the red bars show the histogram of normalized residual values and the reference standard Gaussian distribution as a grey shaded area. In panels **(c)** and **(d)** the fitted distance distribution is given as blue/orange lines as well as the 50 % and 95 % confidence intervals (shaded areas) obtained via the standard method (orange, top) and via bootstrapping (blue, bottom). The ground truth distribution is shown as a shaded grey area for reference. The estimated distribution of the fitted modulation depth λ **(e)** and background decay rate constant κ **(f)** obtained via the standard method are given as orange lines. The kernel density estimations obtained via bootstrapping are given as blue lines. The true values of λ and κ are given as a dashed grey line for reference. All bootstrap results were obtained from 1000 bootstrap samples and the regularization parameter was optimized using AIC for the original signal and fixed throughout the analysis.

To assess the goodness of fit, several procedures can be utilized. A direct test is to compare the histogram of the normalized residuals $|V_{\exp,i}-V_{i}[\theta ]|/\sigma$ to the standard normal distribution N(0,1). Here, σ is the noise standard deviation estimated either from a dataset with several, individually stored scans [Bibr bib1.bibx23], from the standard deviation of the (flat) imaginary part of the signal, or from the residuals of the signal minus the fitted model. Accuracy of the estimate of σ decreases in the sequence indicated, due to a possible imbalance in quadrature channels and the slight model inadequacy that is unavoidable for ill-posed problems. An example of this is shown in Fig. [Fig Ch1.F10]b. If the histogram deviates strongly from a Gaussian distribution, the fit is considered inadequate. Alternatively, the comparison can also be based on a statistical test.

Another method is to examine the reduced $\chi^{2}$-value:

27 $$\chi_{\mathrm{red}}^{2}=\frac{1}{N_{\mathrm{dof}}}\frac{{∥V_{\exp}-V\left[\theta_{\mathrm{fit}}\right]∥}^{2}}{\sigma^{2}}.$$

Here, $N_{\mathrm{dof}}$ is the number of degrees of freedom, which can be taken as approximately equal to the number of data points minus the number of model parameters. A good fit is characterized by $\chi_{\mathrm{red}}^{2}\approx 1$. Note that the use of $\chi_{\mathrm{red}}^{2}$ for non-linear models is not rigorous. Also, the notion of (effective) number of parameters for a non-parametric distribution model, estimated as $\mathrm{tr}(K\bar{K})$ with $\bar{K}$ defined in Eq. ([Disp-formula Ch1.E11]), is not straightforward [Bibr bib1.bibx23].

Additional methods for assessing goodness of fit are discussed in [Bibr bib1.bibx13] and [Bibr bib1.bibx34].

## 9 Uncertainty analysis

Up to this point, we took advantage of knowing the ground truth when we assessed quality of the solutions. However, in experimental work the ground truth is unknown. The presence of noise in experimental signals introduces uncertainty about the underlying noise-free dipolar signal and results in uncertainty about the model parameters. This, in turn, affects the strength of the conclusions that can safely be drawn. For example, $r_{0}=(3.2\pm 0.1)$ nm supports much more confident conclusions about $r_{0}$ than $r_{0}=(3.2\pm 0.9)$ nm. It is therefore crucial to always report uncertainty estimates for all fitted parameters and distance distributions extracted from experimental data, for model parameters $\theta_{i}$ as well as vector elements $P_{j}$ of non-parametric distance distributions. Reporting fitted values without accompanying uncertainty estimates is meaningless. Several approaches have been proposed for uncertainty estimation, including validation of the regularization model [Bibr bib1.bibx47], iterative scanning of the $\chi^{2}$-surface [Bibr bib1.bibx9], covariance matrices [Bibr bib1.bibx77], and Bayesian inference [Bibr bib1.bibx23].

DeerLab provides two separate methods for uncertainty estimation for both model parameters $\theta_{i}$ and for vector elements $P_{j}$ of non-parametric distance distributions.

The first method estimates parameter uncertainties from the covariance matrix [Bibr bib1.bibx13]. For a fully parametric model with parameter vector θ, this is well established [Bibr bib1.bibx38]. The covariance matrix for θ is

28 $$\Sigma_{\theta }=\sigma^{2}{\left(J^{T}J\right)}^{-1},$$

where J is the Jacobian matrix of derivatives with elements $J_{ij}=\partial V_{j}[\theta ]/\partial \theta_{i}$ evaluated at $\theta =\theta_{\mathrm{fit}}$ and is calculated using numerical differentiation.

There is also a simple way to obtain the covariance matrix $\Sigma_{P}$ for the elements of a non-parametric P determined via regularization, but only if the non-negativity constraint for P is disregarded. It is obtained by propagating the time-domain noise covariance matrix $\sigma^{2}I$ (see Eq. [Disp-formula Ch1.E8]) to the distance domain by [Bibr bib1.bibx85]

29 $$\Sigma_{P}=\sigma^{2}\bar{K}{\bar{K}}^{T},$$

with $\bar{K}$ defined in Eq. ([Disp-formula Ch1.E11]). Note that this can be utilized even for models that depend on other parameters, since V always depends linearly on P, as shown above.

From the covariance matrices in Eqs. ([Disp-formula Ch1.E28]) and ([Disp-formula Ch1.E29]), the standard errors of a parameter $\theta_{i}$ or a distribution vector element $P_{i}$ are obtained as

$$\sigma_{\theta_{i}}=\sqrt{(\Sigma_{\theta }{)}_{ii}}$$

and

30 $$\sigma_{P_{i}}=\sqrt{(\Sigma_{P}{)}_{ii}}.$$

These are used to estimate symmetric confidence intervals (CIs) around the fitted parameter for a confidence level γ with boundaries

$$\theta_{\mathrm{fit},i}\pm z_{\gamma }\sigma_{\theta_{i}}$$

and

31 $$P_{\mathrm{fit},i}\pm z_{\gamma }\sigma_{P_{i}},$$

where $z_{\gamma }$ is the γ-quantile of a standard normal or Student's t-distribution.

This method for estimating CIs is simple and stands on a sound theoretical basis. However, it has several limitations. (1) It approximates the parameter likelihood by a Gaussian distribution centered around the fitted $\theta_{\mathrm{fit}}$ and $P_{\mathrm{fit}}$. While this is often a reasonable approximation, it can still fail in many cases. (2) It assumes that the parameters are unbounded. This is not fulfilled in the analysis of dipolar signals, as most parameters are constrained to a certain range, e.g., 0≤λ≤1 and $P_{i}\geq 0$. Although the boundary conditions can be imposed by cropping the CIs in Eq. ([Disp-formula Ch1.E31]) to the parameter range [Bibr bib1.bibx84], the calculation of the CIs is still based on an unbounded assumption. Hence, the CIs do not include the additional information provided by the constraints. (3) In the case of a model that depends on both parameters θ and a non-parametric distribution P, the covariances between $\theta_{i}$ and $P_{j}$ are not accounted for, potentially leading to underestimation of uncertainties.

A more general and accurate method for the estimation of parameter uncertainty involves bootstrapping [Bibr bib1.bibx25]. This is a Monte Carlo resampling method based on generated synthetic signals with different noise realizations. The variant implemented in DeerLab first generates N synthetic traces $V_{k}$ by adding N different noise realizations to the fitted model $V[\theta_{\mathrm{fit}},P_{\mathrm{fit}}]$. The noise is drawn from a Gaussian distribution with standard deviation σ estimated from the experimental data. Then, the N bootstrap traces $V_{k}$ are analyzed in the same fashion as the original dataset $V_{\exp}$, resulting in N+1 fitted parameter vectors $\theta_{\mathrm{fit},k}$ and distance distributions $P_{\mathrm{fit},k}$. The distributions of the parameter values and the distance distribution vector elements are then taken as approximations of the underlying parameter uncertainty distributions.

The bootstrap method, while costly, has several important advantages over the method based on covariance matrices. All information provided by parameter constraints is included in the estimation. Additionally, model-free estimations of parameter distributions are obtained, without the need to assume a Gaussian distribution. These can be statistically analyzed in multiple ways to quantify the parameter uncertainty. For example, in analogy to above, one can define the confidence interval for a parameter θ with confidence level γ=1-α as

32 $$\left(\theta_{1-\alpha /2},\;\theta_{\alpha /2}\right),$$

where $\theta_{1-\alpha /2}$ and $\theta_{\alpha /2}$ are the (1-α/2)th and (α/2)th percentiles of the bootstrapped θ distribution.

Figure [Fig Ch1.F10] shows an example of parameter uncertainty estimation using both the standard method and bootstrap. Although both methods lead to similar confidence intervals, the bootstrapped solution provides narrower intervals thanks to the use of the additional information provided by parameter boundaries and the non-negativity constraint P≥0. While the standard method provides an easily accessible uncertainty estimation, the use of bootstrapping is recommended for producing final results.

While the distance distribution and its uncertainty analysis provide the complete information of the data, in application work it is often of interest to determine summarizing quantities such as the distance mode, the mean distance, or the standard deviation of the distance distribution. It is important to report uncertainties for these quantities as well. If the uncertainty analysis is based on covariance matrices, these uncertainties can be calculated from the covariance matrices $\Sigma_{P}$ and $\Sigma_{\theta }$ using error propagation via the Jacobian. For instance, the variance of the mean distance $\bar{r}$ is

33 $$\sigma_{\bar{r}}^{2}=J\left(\begin{array}{cc} \Sigma_{P} & 0\\ 0 & \Sigma_{\theta } \end{array}\right)J^{T},$$

where J is the Jacobian containing the derivatives of $\bar{r}$ with respect to all model parameters (θ and P). Note that this method does not work for the distance mode. In general, it is preferable to use the more accurate bootstrap method to determine the distance mode, mean, median, and similar quantities. The means and uncertainties of these quantities are easily calculated from their histograms obtained from the ensembles of fitted θ and P.

## 10 Model comparison

The above uncertainty analysis is performed under the assumption of a specific model for the distance distribution (parametric or non-parametric), the background, and the experiment. All estimated uncertainties capture variability only within this assumed model. The possibility that the data could be explained equally well, or better, by other models is not incorporated.

Model selection approaches, as outlined above for parametric and non-parametric distance distribution (selection of number of Gaussians, regularization parameter selection), provide convenient quantitative decision criteria for picking one distribution model over another in a principled fashion. On the other hand, background models are often chosen ad hoc. Finally, the choice of the standard DEER experiment model in Eq. ([Disp-formula Ch1.E3]) is based on physical assumptions (no orientation selection, no bandwidth limitations, no conformer-dependent phase memory times, no exchange couplings, etc.) that might not all be fully valid for a given experimental situation. Whether principled or ad hoc, any model selection eliminates model uncertainty from the analysis and leads to bias.

It is therefore preferable to compare and report the relative performance of a series of plausible models, without picking a winner. This can be accomplished using Akaike weights, defined as [Bibr bib1.bibx14]

34 $$w_{\mathrm{AIC},i}=\frac{e^{-\Delta_{\mathrm{AIC},i}/2}}{\sum_{k=1}^{M}e^{-\Delta_{\mathrm{AIC},k}/2}},$$

where $\Delta_{\mathrm{AIC},i}$ is the difference between the AIC value of model i and the lowest AIC value within the set of models. The Akaike weights give the probability that model i is the best among the set of M candidate models, given the data. This can be used to compare a set of different parametric models or to compare a series of non-parametric distribution models differing in the regularization parameter α.

Figure [Fig Ch1.F11]a illustrates this for a set of parametric models differing in complexity (number of components) and type of basis function. The analysis finds Akaike weights of about 25 % for the 2-Gauss, 3-Gauss and 1-skewed Gauss models, indicating that the data do not provide enough evidence to clearly identify a best model. Figure [Fig Ch1.F11]b shows a comparison for non-parametric distribution models obtained with different α-values, where the AIC is calculated using $\mathrm{tr}(K\bar{K})$ ([Bibr bib1.bibx24]). It is apparent that models over a range of α-values are similarly likely. Therefore, in neither case is there a clear “winner” model. Note that uncertainty in the regularization parameter α could also be propagated to the resulting distance distribution and thereby included in the confidence bands. In cases where this is not applicable, and depending on the conclusions that one wants to draw, it may be prudent to list all models that fit the data reasonably well as opposed to picking the model with the highest weight.

**Figure 11 Ch1.F11:**
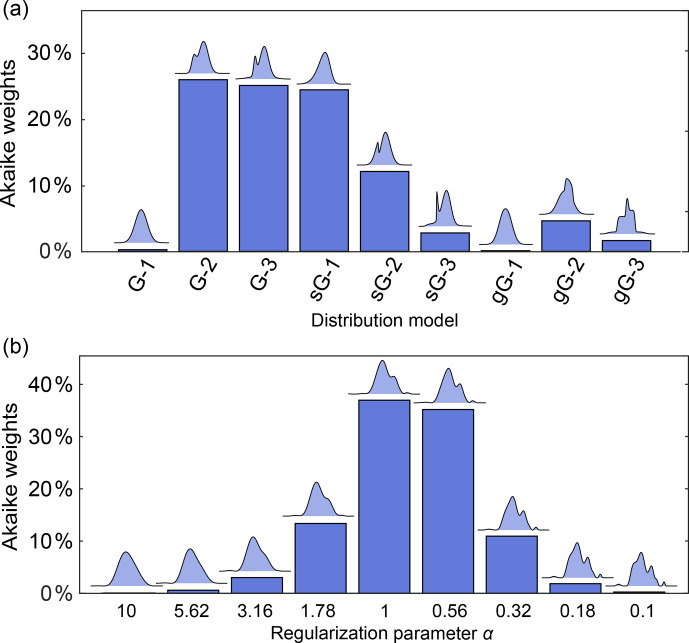
Model comparison using Akaike weights. The Akaike weights (in percentages) are given for **(a)** a set of parametric distance distribution models with varying number n of ordinary (G-n), skewed (sG-n) and generalized (gG-n) Gaussians and **(b)** for non-parametric distance distributions determined over a set of values for the regularization parameter α. For each model in panels **(a)** and **(b)**, the corresponding distance distribution is shown above the bar. Panels **(a)** and **(b)** are based on different simulated DEER signals of different distributions.

Despite these simple numerical procedures for model comparison, researchers need to use careful judgment in which models are included in a comparison and explicitly disclose the reasoning behind all model choices. Also, if the correct model is not included, then even such model comparison will lead to biased or incorrect conclusions.

## 11 Concluding remarks

Dipolar EPR spectroscopy requires reliable and robust data processing tools. The associated software should also be flexible and adaptable to quickly incorporate new developments in the field. DeerLab collects many existing as well as several novel data analysis methods in a flexible, robust, and reliable manner.

By allowing analysis workflows to be shared, DeerLab provides a significant step towards solving reproducibility issues plaguing dipolar EPR spectroscopy analysis. With DeerLab, the analysis scripts can be provided along with published data, leading to improved reproducibility. Through an online repository (see “Code availability” below), all DeerLab versions remain available, further enhancing reproducibility.

DeerLab can serve as a powerful method development tool. It allows the discovery and testing of new processing techniques. We illustrated this by introducing several extensions: one-step analysis with non-parametric distributions, the dipolar multi-pathway model, global analysis with non-parametric distribution models, and uncertainty estimation using bootstrap. The discussion of new methods or comparisons between established ones based on statistical arguments is also largely facilitated by such a scriptable tool.

Although DeerLab does not come with a GUI, it can serve as the data processing engine for intuitive and dedicated GUI-based data analysis tools. These are essential for the robust and successful application of routine dipolar EPR spectroscopy in fields such as structural biology or materials science.

In conclusion, DeerLab provides a unified and extensible open-source platform for data analysis in dipolar EPR spectroscopy, opening up a new world of data processing workflows.

## Supplement

10.5194/mr-1-209-2020-supplement All DeerLab (version 0.10.0) scripts employed for generating the figures in this work are available in the Supplement. The supplement related to this article is available online at: https://doi.org/10.5194/mr-1-209-2020-supplement.

## Code availability

The version of the DeerLab source code used for this paper is available at http://doi.org/10.5281/zenodo.4058605. Further information, examples, and documentation can be found there.
