# Supplementary material for: DeerLab: a comprehensive software package for analyzing dipolar electron paramagnetic resonance spectroscopy data
Source: Magn Reson (Gott). 2020 Oct 1;1(2):209–24. doi: 10.5194/mr-1-209-2020 (PMC8462493; doi:10.5194/mr-1-209-2020)
Supplement: All DeerLab (version 0.10.0) scripts employed for generating the figures in this work are available in the Supplement. The supplement related to this article is available online at: https://doi.org/10.5194/mr-1-209-2020-supplement. [file mr-1-209-supplement.zip › mr-1-209-2020-supplement-title-page.pdf]

Supplement of Magn. Reson., 1, 209–224, 2020
https://doi.org/10.5194/mr-1-209-2020-supplement
© Author(s) 2020. This work is distributed under
the Creative Commons Attribution 4.0 License.

Open Access
MAGNETIC
RESONANCE

Supplement of

DeerLab: a comprehensive software package for analyzing dipolar electron paramagnetic resonance spectroscopy data

Luis Fá

Correspondence to: Stefan Stoll (stst@uw.edu)

- mr-1-209-2020-supplement-title-page.pdf
- DeerLab_SI-mr-2020-13.pdf
- scripts
  - data
    - figure10_Pdata.txt
    - figure10_Vdata.txt
    - figure1a_Pdata.txt
    - figure1a_Vdata.txt
    - figure1b_Pdata.txt
    - figure1b_Vdata.txt
    - figure2_Pdata.txt
    - figure2_Vdata.txt
    - figure4_Pdata.txt
    - figure4_Vdata.txt
    - figure6a_Pdata.txt
    - figure6a_Vdata.txt
    - figure6b_Pdata.txt
    - figure6b_Vdata.txt
    - figure7_Pdata.txt
    - figure7_Vdata.txt
    - figure8_Pdata.txt
    - figure8_Vdata.txt
  - figure1_regularization.m
  - figure1_regularization.py
  - figure2_multigauss.m
  - figure2_multigauss.py

- figure4_onestepanalysis.m
- figure4_onestepanalysis.py
- figure6_multipathway.m
- figure6_multipathway.py
- figure7_globalfit.m
- figure7_globalfit.py
- figure8_modelfree_titration.m
- figure8_modelfree_titration.py
- figure_10_uncertaintyestimation.m
- figure_10_uncertaintyestimation.py
- groundtruth.m

The copyright of individual parts of the supplement might differ from the CC BY 4.0 License.
