# Supplementary material for: DeerLab: a comprehensive software package for analyzing dipolar electron paramagnetic resonance spectroscopy data
Source: Magn Reson (Gott). 2020 Oct 1;1(2):209–24. doi: 10.5194/mr-1-209-2020 (PMC8462493; doi:10.5194/mr-1-209-2020)
Supplement: All DeerLab (version 0.10.0) scripts employed for generating the figures in this work are available in the Supplement. The supplement related to this article is available online at: https://doi.org/10.5194/mr-1-209-2020-supplement. [file mr-1-209-supplement.zip › DeerLab_SI-mr-2020-13.pdf]

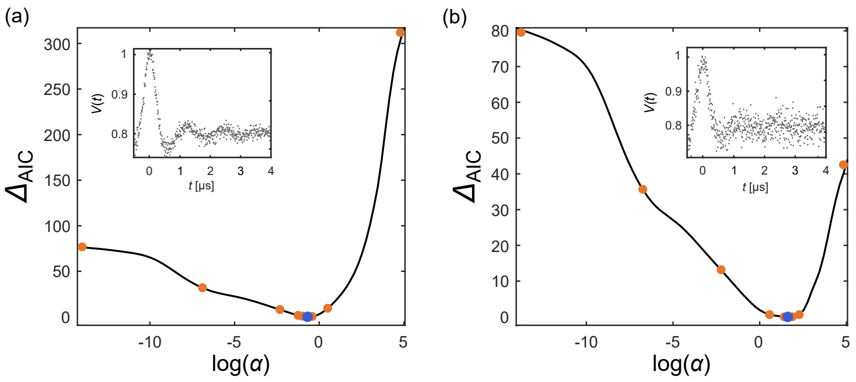

Figure S1: Selection of the optimal regularization parameter α using the Akaike information criterion (AIC) for the examples in Fig. 2. The black solid line represents the AIC functional and the orange dots represent the evaluated α-values during a golden-search of the AIC functional. The optimal value of α is represented as a blue dot. The dipolar signals used for the examples in Fig. 1 are given as insets.

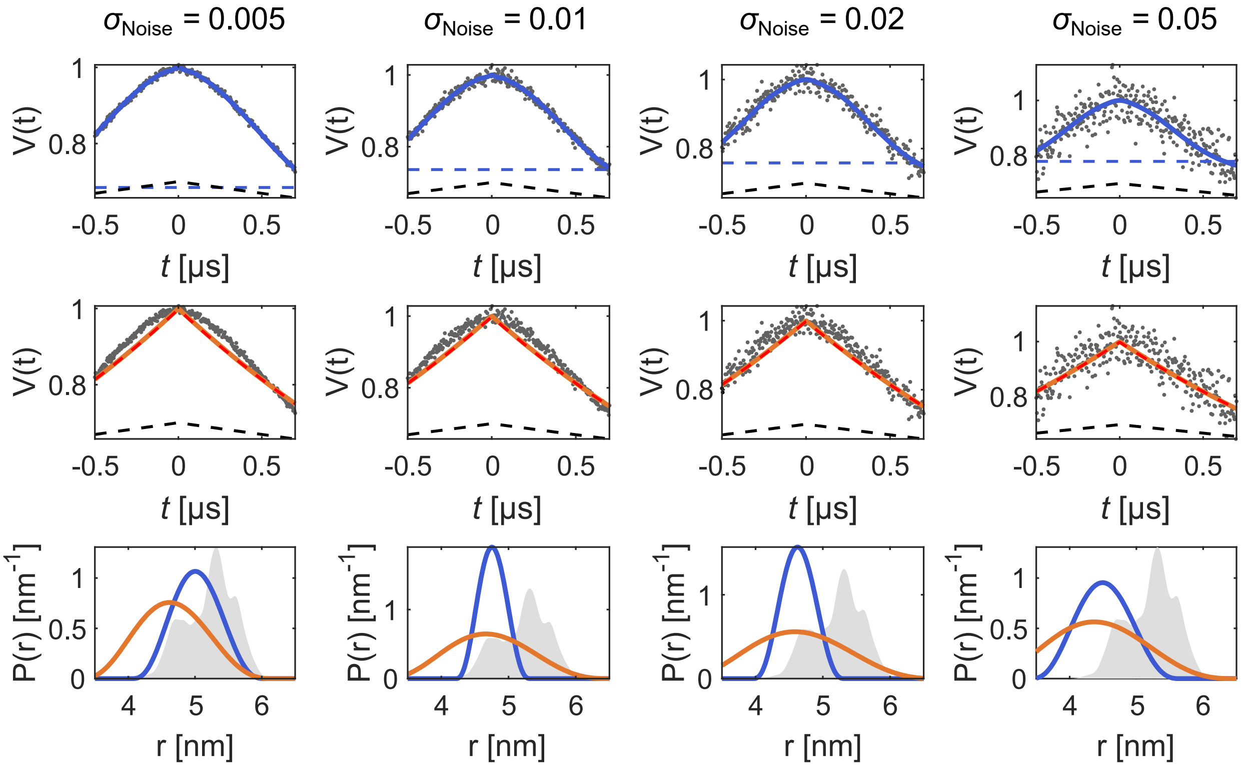

Figure S2: Effect of noise on the analysis of highly truncated dipolar signals. The most truncated signal in Fig. 4d of the main text is analyzed at different noise standard deviations specified for each column as σ_{noise}. The analysis was done by either fitting both simultaneously (blue) or by fitting the background followed by the distribution (two-step analysis, orange). The data is given as grey dots, and the fitted signal and background are given as solid and dashed lines, respectively. The true background is given as a grey dashed line for reference. The non-parametric distance distributions obtained via Tikhonov regularization are given as respectively colored lines and the ground truth as a shaded area.

1 DeerLab scripts

All DeerLab scripts and associated data used for the figures in the main text are provided along this SI. These were written using DeerLab (release 0.10.0) in Python 3.6 (Windows 64bit).

Figure	Script	Data files
Fig. 1	figure1_regularization.py	figure1a_Pdata.txt, figure1a_Vdata.txt, figure1b_Pdata.txt, figure1b_Vdata.txt
Fig. 2	figure2_multigauss.py	figure2_Pdata.txt, figure2_Vdata.txt
Fig. 4	figure4_onestepanalysis.py	figure4_Pdata.txt, figure4_Vdata.txt
Fig. 6	figure6_multipathway.py	figure6a_Pdata.txt, figure6a_Vdata.txt, figure6b_Pdata.txt, figure6b_Vdata.txt
Fig. 7	figure7_globalfit.py	figure7_Pdata.txt, figure7_Vdata.txt
Fig. 8	figure8_modelfree_titration.py	figure8_Pdata.txt, figure8_Vdata.txt
Fig. 10	figure_10_uncertaintyestimation.py	figure10_Pdata.txt, figure10_Vdata.txt

All distributions are based on the large DEER data library simulated by Edwards and Stoll (2018) from a T4 lysozyme structure (available here). Since the distances in the library are on average short, the distributions in our examples were generated by extracting the distributions shapes from the library and interpolating them on a new distance axis.
